# Hepatocellular carcinoma subtypes based on metabolic pathways reveals potential therapeutic targets

**DOI:** 10.3389/fonc.2023.1086604

**Published:** 2023-03-02

**Authors:** Zehua He, Qingfeng Chen, Wanrong He, Junyue Cao, Shunhan Yao, Qingqiang Huang, Yu Zheng

**Affiliations:** ^1^ College of Life Science and Technology, Guangxi University, Nanning, Guangxi, China; ^2^ School of Computer, Electronic and Information, Guangxi University, Nanning, Guangxi, China; ^3^ Department of Gastroenterology, People’s Hospital of Guangxi, Zhuang Autonomous Region, Nanning, Guangxi, China; ^4^ Medical College, Guangxi University, Nanning, Guangxi, China; ^5^ Guigang City Department of Radiology, People’s Hospital, Guigang, Guangxi, China; ^6^ Department of Computer Science and Information Technology, La Trobe University, Melbourne, VIC, Australia

**Keywords:** hepatocellular carcinoma (HCC), metabologenomics, bioinformatics, microenvironment, targeted therapy

## Abstract

**Introduction:**

Hepatocellular carcinoma (HCC) is an aggressive malignancy with steadily increasing incidence rates worldwide and poor therapeutic outcomes. Studies show that metabolic reprogramming plays a key role in tumor genesis and progression. In this study, we analyzed the metabolic heterogeneity of epithelial cells in the HCC and screened for potential biomarkers.

**Methods:**

The hepatic single-cell RNA sequencing (scRNA-seq) datasets of HCC patients and healthy controls were obtained from the Gene Expression Omnibus (GEO) database. Based on data intergration and measurement of differences among groups, the metabolic epithelial cell subpopulations were identified. The single-cell metabolic pathway was analyzed and the myeloid subpopulations were identified. Cell-cell interaction analysis and single-cell proliferation analysis were performed. The gene expression profiles of HCC patients were obtained from the GSE14520 dataset of GEO and TCGA-LIHC cohort of the UCSC Xena website. Immune analysis was performed. The differentially expressed genes (DEGs) were identified and functionally annotated. Tumor tissues from HCC patients were probed with anti-ALDOA, anti-CD68, anti-CD163, anti-CD4 and anti-FOXP3 antibodies. Results We analyzed the scRNA-seq data from 48 HCC patients and 14 healthy controls. The epithelial cells were significantly enriched in HCC patients compared to the controls (p = 0.011). The epithelial cells from HCC patients were classified into two metabolism-related subpopulations (MRSs) – pertaining to amino acid metabolism (MRS1) and glycolysis (MRS2). Depending on the abundance of these metabolic subpopulations, the HCC patients were also classified into the MRS1 and MRS2 subtype distinct prognoses and immune infiltration. The MRS2 group had significantly worse clinical outcomes and more inflamed tumor microenvironment (TME), as well as a stronger crosstalk between MRS2 cells and immune subpopulations that resulted in an immunosuppressive TME. We also detected high expression levels of ALDOA in the MRS2 cells and HCC tissues. In the clinical cohort, HCC patients with higher ALDOA expression showed greater enrichment of immunosuppressive cells including M2 macrophages and T regulatory cells.

**Discussion:**

The glycolytic subtype of HCC cells with high ALDOA expression is associated with an immunosuppressive TME and predicts worse clinical outcomes, providing new insights into the metabolism and prognosis of HCC.

## Introduction

Hepatocellular carcinoma (HCC) is an aggressive malignancy with high incidence rates worldwide and poor therapeutic outcomes (1). Since most HCC patients are diagnosed at the advanced stage, chemotherapy is the recommended treatment regimen (2). However, conventional systemic chemotherapy has negligible clinical benefits. In fact, doxorubicin, doxorubicin plus sorafenib, and the FOLFOX4 regimen (fluorouracil, oxaliplatin and leucovorin (folinic acid)) have failed to improve the survival rates of HCC patients and are also associated with considerable toxicity (3, 4). Furthermore, small-molecule tyrosine kinase inhibitors (TKIs), the first/second-line drugs for HCC approved by U.S. Food and Drug Administration (FDA), also have limited clinical benefits due to frequent development of resistance (5). On the other hand, immunotherapy has achieved significant survival benefits for HCC patients; a recent phase III trial reported median overall survival (OS) of 19.2 months in the atezolizumab-bevacizumab arm compared to 13.4 months in the sorafenib arm (6). Nevertheless, there is an urgent need to explore effective novel strategies for the treatment of HCC.

Metabolic reprogramming is a notable hallmark of cancer (7) that fulfills the biomass and energy demands of the rapidly proliferating cells during tumor initiation and progression (8, 9). Unlike normal cells, tumor cells preferentially use glycolysis to meet the energy needs for proliferation, invasion and metastasis (10–12). Recent studies have shown that remodeling of lipid metabolism is essential for the proliferation and malignant transformation of hepatocytes during HCC progression (13–15). Hall et al. (13) reported an altered lipid signature in human HCC cells, and showed a positive correlation between monounsaturated phosphatidylcholine and hepatic carcinogenesis. However, (16) found that the mitochondrial protein LACTB inhibited the proliferation and differentiation of tumor cells by altering lipid metabolism (13). These contraindicatory findings suggest that metabolic reprogramming plays a far more complex role in tumorigenesis than previously believed, and should be explored as a novel target for inhibiting tumor growth and overcoming drug resistance.

The tumor microenvironment (TME) is a critical factor in tumor progression. Previous studies have shown that the interaction between tumor cells and the immune or stromal cells in the TME promote tumor development and progression (17–19), leading to the poor clinical outcomes. In addition, the metabolic characteristics of different immune cells and stromal cells are promising biomarkers of tumor initiation and progression (20, 21). However, given the intra-tumoral heterogeneity, and the crosstalk between tumor cells and immune or stromal cells in the microenvironment, the extent of metabolic reprogramming in the TME is unclear. Thus, exploring the metabolic heterogeneity and diversity in HCC and other tumors can help identify potential targets for personalized treatment.

To this end, we performed integrated single-cell transcriptomics analysis of tumor samples from HCC patients in order to dissect the metabolic phenotypes of cells in the HCC microenvironment. We were able to define two metabolic subtypes of HCC with distinct immunological characteristics and clinical outcomes, and identified the biomarkers that can distinguish between these metabolic subtypes. Our findings offer new insights into the metabolism and prognosis of HCC, which can improve the diagnostic accuracy and therapeutic outcomes.

## Materials and methods

### Acquisition and pre-processing of scRNA-seq datasets

The hepatic single-cell RNA sequencing (scRNA-seq) datasets of HCC patients and healthy controls, including GSE112271 (22), GSE149614 (23), GSE151530 (24) and GSE156625 (25), were obtained from the Gene Expression Omnibus (GEO) database. Quality control and pre-processing procedures were performed using Seurat (4.0.5, https://satijalab.org/eurat/) R toolkit (26). To avoid the influence of abnormal cells and technical noise on downstream analysis, low-quality cells such as doublets and empty droplets were removed. In addition, cells with mitochondrial gene expression >10%, or number of detected genes < 200 or > 5000 were also removed. Samples with > 1000 cells were retained for the analysis. Finally, the data of 165,932 cells, including 126,345 cells from 48 HCC patients and 39,587 cells from 14 healthy donors, were used for further analysis.

### Data integration

In order to minimize the technical batch effects among individuals and experiments, the “RunHarmony” function in R package harmony (27) was used to integrate all cells from HCC patients and healthy donors. R package “Seurat” was used for principal component analysis (PCA) and dimensionality reduction. The top 4000 variable genes were used for PCA to reduce dimensionality. The dimensionality of the scaled and integrated data matrix was further reduced to two-dimensional space based on the first 30 principal components (PCs), and visualized by t-Distributed Stochastic Neighbor Embedding (tSNE). The cell clusters were identified based on a shared nearest neighbor (SNN) modularity optimization-based clustering algorithm with a resolution of 1. According to the expression levels of some well-known markers (24), the cells were annotated as B cells, T/NK cells, myeloid cells, fibroblasts, endothelial cells, and epithelial cells.

#### Measurement of differences among samples

To measure the differences among epithelial cells from all HCC and healthy control samples, the expression profile was scaled using the “scale” function. The “cor” function was then used to calculate the correlation between samples (-1 to 1). The correlation distance between any two samples was defined as “1-correlation”, which ranged from 0 (highly identical) to 2 (completely different).

### Identification of metabolic epithelial cell subpopulations

To identify the metabolic subpopulations of epithelial cells from HCC patients, the cell were first reintegrated using RunHarmony (27) and then classified into 42 clusters in an unsupervised manner using the first 30 PCs and the resolution of 2. The mean expression values of metabolic genes in these cells (28) were downloaded from Kyoto Encyclopedia of Genes and Genomes (KEGG), and was calculated for each cluster. Consensus clustering was then performed to determine the optimal number of stable metabolism-related epithelial subpopulations for HCC according to the expression matrix of metabolic genes for 42 clusters using the “ConsensusClusterPlus” package in R (1000 iterations, 80% resampling). he 42 clusters were divided into two metabolism-related subpopulations (MRS1 and MRS2), and the “FindAllMarkers” function was applied to identify the specific genes for each MRS, with log2 fold-change (avg_log2FC) > 0.25, detectable expression in at least 25% of the cells and percentage ratio > 1 as the criteria. The top 50 genes with higher avg_log2FC for each MRS were designated as the respective MRS1 and MRS2 genes.

### Single-cell metabolic pathway analysis

The single-sample gene sets enrichment analysis (ssGSEA) scores of 85 KEGG metabolic pathways was calculated for each cluster based on the gene expression level in each cell (29). The differentially activated pathways between MRS1 and MRS2 clusters were then identified by Wilcoxon rank-sum test with p < 0.05 as the cut-off.

### Identification of myeloid subpopulations

The myeloid cells were reintegrated using RunHarmony (27) and classified into 22 unsupervised clusters using the first 20 PCs and resolution of 0.8. The specific genes for each cluster were identified using FindAllMarkers with expression percentage ≥ 0.25 and avg_logFC ≥ 0.25 as the criteria. Based on established biomarkers, the 22 clusters were classified into eight major subpopulations.

### Cell-cell interaction analysis

The molecular interaction networks between the epithelial MRSs and myeloid subpopulations for HCC patient were identified using CellPhoneDB (30). The ligand-receptor pairs with p value < 0.05 were screened for the different cell clusters.

### Single-cell proliferation analysis

Single cell proliferation was estimated by predicting the cell cycle distribution using the “CellCycleScoring” function, which is based on the expression levels of ten genes that are upregulated in cycling cells (ASPM, CENPE. CENPF, DLGAP5, MKI67, NUSAP1, PCLAF, STMN1, TOP2A, TUBB) (31). For each of these genes, a background set of 100 genes with the smallest difference in average expression levels was selected. The average expression of the background gene set was then subtracted from each signature gene, and the average of the resulting values was calculated as the proliferation score. To identify metabolic subgroups of HCC patients at bulk levels, the MRS1 and MRS2 scores for HCC patients in TCGA-LIHC and GSE14520 cohort were calculated by GSEA in R package “GSVA” (32) based on the expression levels of the MRS1 and MRS2 genes at single-cell level. The samples were then assigned to the MRS1 and MRS2 subgroups on the basis of the respective scores.

### Metabolic subgrouping for HCC patients

The gene expression profiles of HCC patients were obtained from the GSE14520 (33) dataset of GEO (https://www.ncbi.nlm.nih.gov/geo/) and TCGA-LIHC cohort of the UCSC Xena website (https://xenabrowser.net/datapages/). The robust multi-array average (RMA) algorithm in the affy package was used to pre-process the array profiles. After background correction, quantile normalization and probe summarization, the gene expression profile was generated based on the platform providing gene and probe mappings. Samples with overall survival (OS) above zero-days were selected for further analysis.

### Immune analysis

The tumor purity and stromal/immune infiltration were calculated for each sample based on bulk transcriptomic profile using the ESTIMATE algorithm (34). The tumor purity, ESTIMATE score, immune score and stromal score were calculated. In addition, the signature genes of 29 immune cell types and immune-related pathways were obtained from a previous study (35), and the abundance of these signatures in each patient was estimated by ssGSEA using the R package GSVA (32). Since the anti-cancer immune response determines the fate of tumor cells (36), the specific signatures associated with each immune response pathway were obtained from a previous study (37) and ssGSEA was performed.

#### Identification and functional annotation of differentially expressed genes (DEGs)

For single-cell datasets, the FindAllMarkers function was used to identify the specific genes of each group. For bulk population datasets, the R package “limma” (38) was used to screen DEGs with adjusted P-value < 0.05 and |FC| ≥ 2 as the thresholds. Gene Ontology (GO) and KEGG pathway enrichment analyses were performed using the R package clusterProfile. The top ten enriched GO and KEGG pathways were displayed.

#### Immunohistochemistry and immunofluorescence

Tumor tissues from HCC patients were probed with anti-ALDOA (DF3068, Affinity), anti-CD68 (66231-2-Ig, Proteintech), anti-CD163 (16646-1-AP, Proteintech), anti-CD4 (67786-1-Ig, Proteintech) and anti-FOXP3 (BA2032-1, Wuhan Boster Biologicals) antibodies. The sections were observed with the image acquisition system of OLYMPUS UC90 (Japan) and the positively stained regions and the number of positive cells were analyzed using Image-Pro Plus (Media Cybernetics, USA).

### Statistical analysis

All statistical analyses were performed using R software (version 4.0.4). The continuous variables between two groups were compared using the Wilcoxon rank-sum test, and Fisher’s exact test was used to compare the categorical variables. The prognostic factors were identified using the log-rank test. All tests were two-tailed and p-value ≤ 0.05 was considered statistically significant.

## Results

### The HCC microenvironment is enriched in epithelial cells

**Figure 1:**
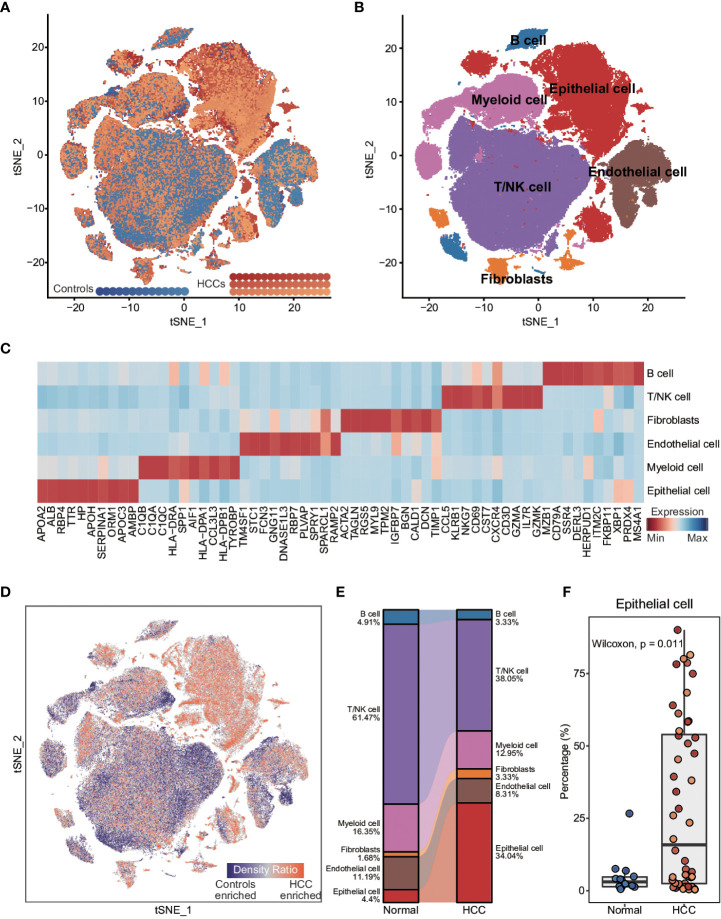
Comparison of cellular components between HCC patients and healthy controls at single-cell resolution **(A)** t-distributed stochastic neighbor embedding (t-SNE) visualization of 165,932 cells from 48 HCC patients and 14 healthy controls. **(B)** t-SNE visualization of cell types annotated by classical gene markers. **(C)** Heatmap showing the top ten cell type-specific genes identified by the FindAllMarkers function. **(D)** Heatmap showing the distribution density of cells from HCC patients and healthy controls. The UMAP visualization is split into 200×200 bins. **(E)** Sankey plot showing the difference in cellular states between HCC patients and healthy controls. **(F)** Boxplot showing the proportion of cell types in HCC patients and healthy controls. Wilcoxon rank-sum test was used to measure the differences between two groups. Horizontal lines in the boxplots represent the median, the lower and upper hinges correspond to the first and third quartiles, and the whiskers extend from the hinge up to 1.5 times the interquartile range from the hinge.

To explore the inter- and intra-tumoral heterogeneity of HCC at the single-cell level, we analyzed the scRNA-seq data from 48 HCC patients and 14 healthy controls. As shown in **Figure 1A**, a total of 165,932 individual cells from nine samples were clustered into six major cell types. The clusters were explored by PCA and visualized by t-distributed stochastic neighbor embedding (t-SNE). The cells in each cluster were annotated with the canonical markers (**Figures 1B, C**; **Supplementary Figure 1A**, and **Supplementary Table 1**) as B cells (6150), endothelial cells (14,926), epithelial cells (44,745), fibroblasts (4871), myeloid cells (22,834) and T/NK cells (72,406). We compared the enrichment of these cell types between HCC patients and healthy controls (**Figures 1D, E**), and found that epithelial cells were significantly enriched in HCC patients compared to the controls (p = 0.011; **Figure 1F** and **Supplementary Figure 1B**), whereas the percentage of endothelial cells was significantly lower in HCC patients (p = 0.053; **Supplementary Figure 1B**). In addition, the T/NK and B cells were also considerably reduced in HCC patients (T/NK cells: p = 0.062; B cells: p = 0.025; **Figure 1E** and **Supplementary Figure 1B**), while the percentage of myeloid cells was similar in both groups (p = 0.17; **Figure 1E** and **Supplementary Figure 1B**). These results indicated that epithelial cells and immune cells likely play important roles in the pathogenesis of HCC.

### Stratification of epithelial cells in HCC patients based on metabolism-related genes

Recent studies have shown that metabolic reprogramming is a hallmark of cancer (7). To evaluate possible metabolic reprogramming in the epithelial cells of HCC tumors, we extracted 1679 metabolism-related genes from the KEGG database (28). Consensus clustering and PCA revealed a distinct metabolic gene expression pattern of the epithelial cells from HCC patients compared to the controls, whereas the epithelial cells from the controls samples showed high similarity (**Supplementary Figures 2A, B**). Furthermore, the global shifts in metabolic gene expression between and within epithelial cells from HCC patients and healthy controls were measured by the correlation distance. The distance between epithelial cells from HCC patients and healthy controls or within the cells from HCC patients was significantly greater than that within the cells from healthy controls (**Supplementary Figure 2C**), indicating considerable metabolic heterogeneity among the epithelial cells from HCC patients.

**Figure 2:**
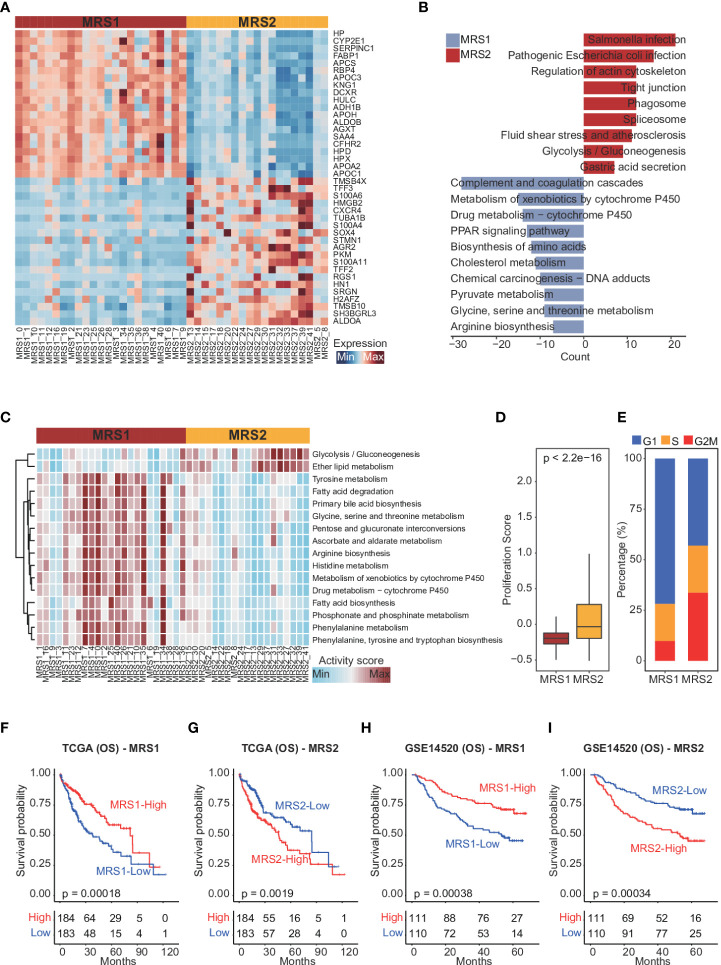
Dissection of metabolic epithelial cell subpopulations from HCC patients **(A)** Heatmap showing the top 20 subpopulation-specific genes identified by the FindAllMarkers function. **(B)** Functional enrichment analysis of subpopulation-specific genes. **(C)** Heatmap of metabolic pathways specifically activated in MRS1 and MRS2. **(D)** Boxplot showing the proliferation scores of MRS1 and MRS2. Wilcoxon rank-sum test was used to measure the differences between two groups. Horizontal lines in the boxplots represent the median, the lower and upper hinges correspond to the first and third quartiles, and the whiskers extend from the hinge up to 1.5 times the interquartile range from the hinge. **(E)** Fraction of cells in the G1 (blue), S (orange) and G2M (red) phases in MRS1 and MRS2. **(F-I)** Kaplan-Meier curves showing the overall survival (OS) of HCC patients in TCGA-LIHC **(F, G)** and GSE14520 **(H, I)** cohorts. All patients were categorized into MRS1 and MRS2 groups based on the median enrichment scores of each.

To confirm this metabolic heterogeneity, we re-clustered the epithelial cells from HCC patients into 42 clusters (**Supplementary Figures 3A, B**), which were then classified into two heterogeneous subpopulations (MRS1 and MRS2) based on metabolism-related genes expression matrix using consensus clustering (**Figure 2A** and **Supplementary Figure 3C**). We also identified the DEGs between MRS1 and MRS2 cells at the single-cell level (**Figure 2A** and **Supplementary Table 2**), and their functional enrichment analyses revealed distinct metabolic patterns of the MRS1 and MRS2 cells. As shown in **Figure 2B**, pathways related to the metabolism of glycine, serine, threonine and other amino acids were obviously activated in the MRS1 cells, while glycolysis/gluconeogenesis was significantly activated in MRS2 cells. Furthermore, single-cell metabolic pathway analysis also revealed activation of tyrosine, glycine, serine, threonine, and phenylalanine metabolism, and phenylalanine, tyrosine and tryptophan biosynthesis in MRS1 cells. In contrast, glycolysis and gluconeogenesis were up-regulated in MRS2 cells at the single-cell level (**Figure 2C**). Consistent with the role of glycolysis in supporting tumor cell proliferation (39), the MRS2 cells also showed higher proliferation scores (**Figure 2D**), and more than 50% of these cells were either in S or G2M phase (**Figure 2E**). Taken together, the epithelial cells from HCC patients were classified into two distinct metabolic subtypes, of which the MRS2 cells had significant activation of glycolysis/gluconeogenesis and higher proliferation rates.

### The metabolic phenotypes of epithelial cells are associated with the clinical outcomes of HCC

**Figure 3:**
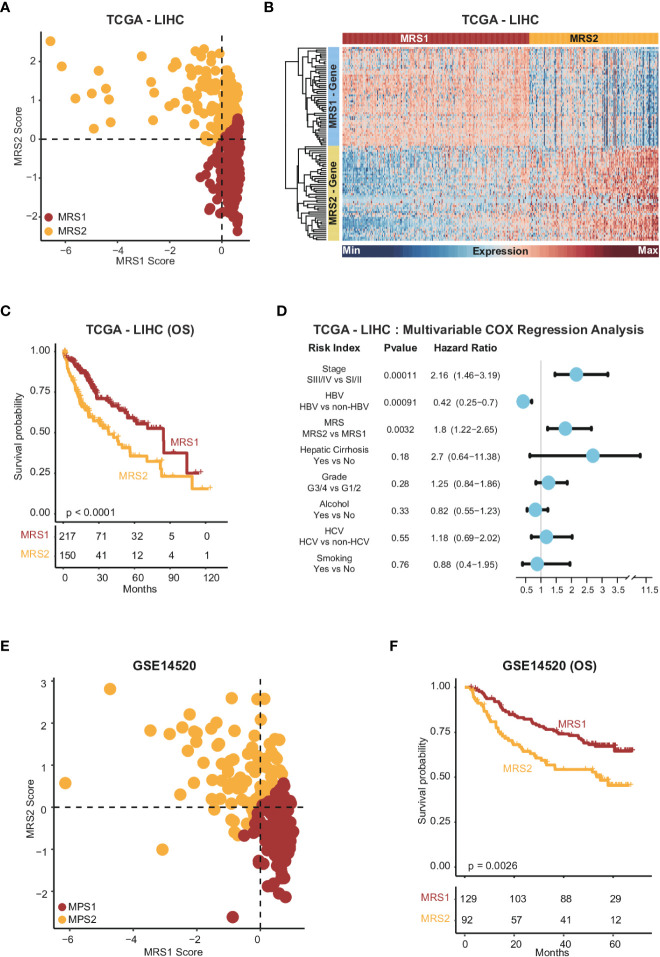
Stratification of HCC samples based on the expression of MRS1 and MRS2 genes **(A)** Scatter plot showing enrichment scores of MRS1 genes (x-axis) and MRS2 genes (y-axis) in each sample in TCGA-LIHC cohort. Metabolic subgroups were assigned based on the enrichment scores of MRS1 and MRS2 genes (see "Methods"). **(B)** Heatmap showing the co-expressed MRS1 and MRS2 genes in two metabolic subgroups in TCGA-LIHC cohort. **(C)** Kaplan-Meier curves showing the OS of HCC patients with MRS1 or MRS2 phenotype in TCGA-LIHC cohort. **(D)** Multivariate COX regression analysis of metabolic subtypes and clinical characteristics in TCGA-LIHC cohort. **(E)** Scatter plot showing enrichment scores of MRS1 genes (x-axis) and MRS2 genes (y-axis) in each sample in GSE14520 cohort. Metabolic subgroups were assigned based on the enrichment scores of MRS1 and MRS2 genes (see "Methods"). **(F)** Kaplan-Meier curves showing OS of HCC patients with MRS1 or MRS2 phenotype in GSE14520 cohort.

To further explore the relationship between the metabolism-related epithelial subpopulations and clinical outcomes, we calculated the MRS1 and MRS2 scores for HCC patients in TCGA-LIHC and GSE1450 cohorts. As shown in **Figures 2F, G**, higher proportion of MRS1 corresponded to favorable overall survival (OS) (log-rank test, p = 0.00018), whereas the predominance of MRS2 correlated to poor OS (log-rank test, p = 0.0019). We validated these results in an independent cohort, and the trend was consistent with the observations in TCGA-LIHC cohort (**Figures 2H, I**). Accordingly, the HCC patients were divided into the MRS1 and MRS2 groups according to the respective enrichment scores (**Figures 3A, B**). As expected, patients in the MRS2 group had significantly wore OS compared to the MRS1 patients (log-rank test, p < 0.001, **Figure 3C**). Furthermore, multivariate COX regression analysis revealed that MRS2 was an independent predictor of worse prognosis in HCC after adjusting for stage, grade, HBV and HCV infection, hepatic cirrhosis and alcohol (**Figure 3D**). In another independent cohort as well, the MRS2 patients showed worse OS compared to the MRS1 patients (**Figures 3E, F**). Taken together, HCC can be divided into two metabolic phenotypes, and the predominance of epithelial cells with enhanced glycolysis/gluconeogenesis is linked to worse outcome.

### Metabolism-related subtypes are associated with distinct immune features

**Figure 4:**
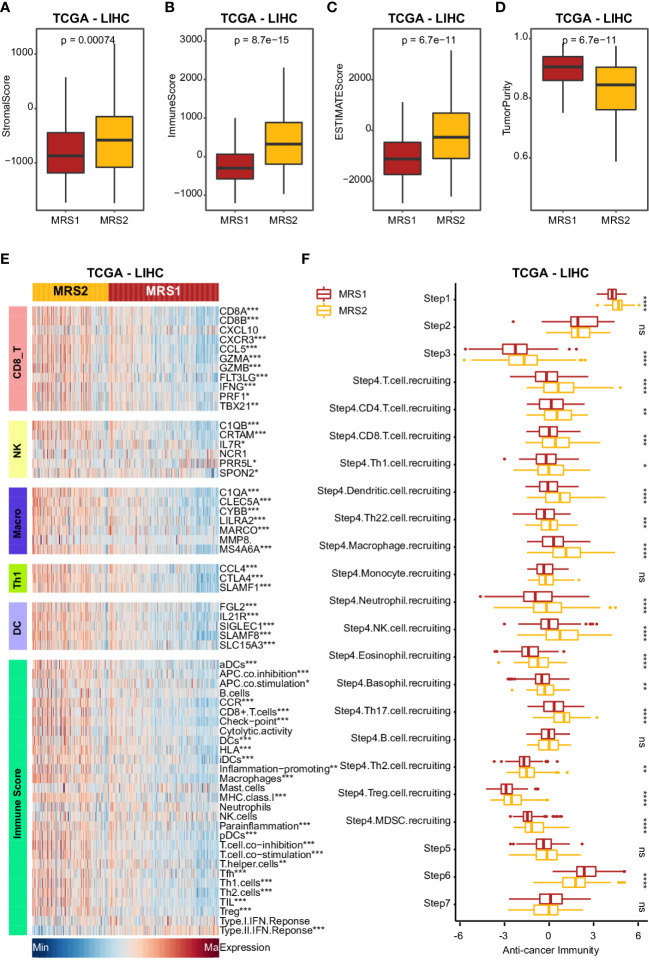
Clinical impact of immunophenotypes in the metabolic subgroups in TCGA-LIHC cohort **(A-D)** Boxplot showing four TME scores calculated using the ESTIMATE algorithm in two subgroups. Wilcoxon rank-sum test was used to measure the differences between two groups. Horizontal lines in the boxplots represent the median, the lower and upper hinges correspond to the first and third quartiles, and the whiskers extend from the hinge up to 1.5 times the interquartile range from the hinge. **(E)** Heatmap comparing immune markers, immune cell infiltration and immune-related response in the two metabolic subgroups. **(F)** Boxplot showing the difference in immune responses between MRS1 and MRS2 in TCGA-LIHC cohort. *p-value < 0.05, **p-value < 0.01, ***p-value < 0.001, ****p-value < 0.0001, and ns p-value ≥ 0.05.

Studies show that the immune cells in the TME can promote tumor development and progression by interacting with tumor cells (40, 41), leading to poor outcomes. Therefore, we next analyzed the differences in various immune-related factors between the two metabolic subtypes. Patients with the MRS2 phenotype exhibited higher ESTIMATE score, immune score and stromal score, which corresponded to lower tumor purity (**Figures 4A–D** and **Supplementary Figures 4A–D**). Furthermore, most immune cell populations, and the activity of immune-related pathways were significantly enriched in HCC patients with the MRS2 phenotype (**Figure 4E** and **Supplementary Figure 4E**). The regulatory T cells (Tregs) and myeloid-derived suppressor cells (MDSCs) that contribute to the immunosuppressive TME (42, 43) were particularly enriched in the MRS2 patients (**Figure 4F** and **Supplementary Figure 4F**). These results suggested that the worse prognosis associated with the MRS2 subtype can be attributed to greater infiltration of Tregs and MDSCs.

### STMN1+ monocyte and macrophages contribute to the immunosuppressive microenvironment of HCC by interacting with MRS2 cells

**Figure 5:**
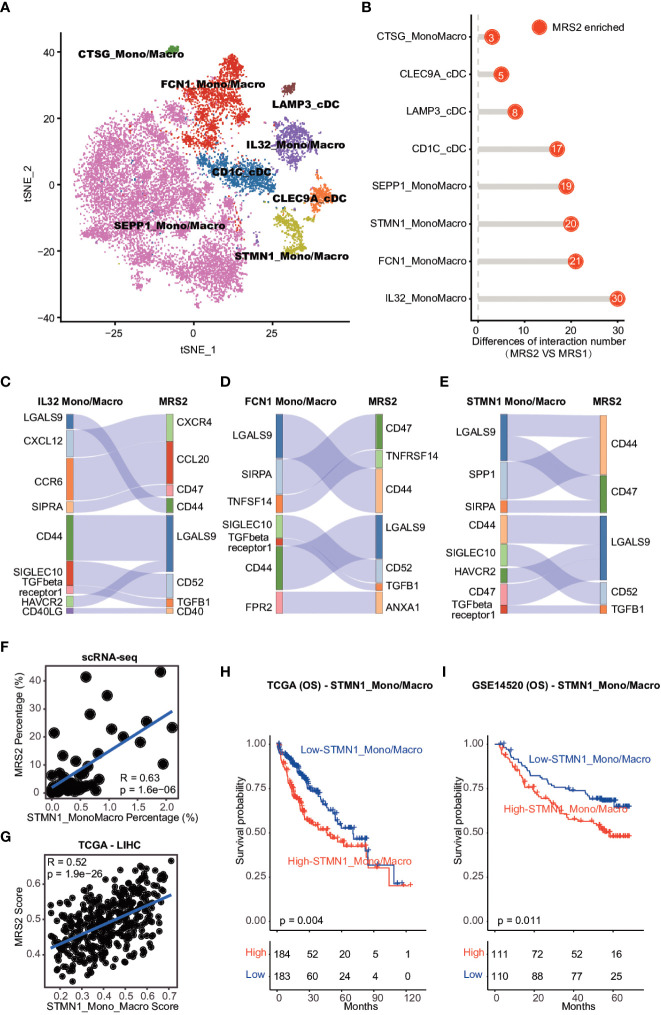
Cell-cell interaction between MRS2 cells and myeloid cells **(A)** t-SNE visualization of eight subpopulations of myeloid cells. **(B)** The difference in the number of ligand-receptor interactions between MRS1 and MRS2 cells. **(C)** Inhibitory interactions between IL32^+^ mono/macrophage and MRS2 cells. **(D)** Inhibitory interactions between FCN1^+^ mono/macrophages and MRS2 cells. **(E)** Inhibitory interactions between STMN1^+^ mono/macrophages and MRS2 cells. **(F)** Correlation between the percentage of STMN1 mono/macrophages and the fraction of MRS2 cells in HCC patients at single-cell level. **(G)** Correlation between the enrichment scores of STMN1^+^ mono/macrophages and MRS2 cells of HCC patients in TCGA-LIHC cohort. **(H, I)** Kaplan-Meier curves showing OS of HCC patients in TCGA-LIHC **(H)** and GSE14520 **(I)** cohorts. All patients were categorized into two groups based on the median of enrichment scores of STMN1^+^ mono/macrophages.

Given the positive correlation observed between the MRS2 epithelial cells and immune cells in HCC (**Supplementary Figures 5A–D**), we validated this result at the single-cell level. While MRS1 cells were not significantly correlated to the different immune cell populations, the MRS2 cells were co-enriched with myeloid cells (**Supplementary Figures 5E–G**). In addition, the percentage of MRS2 cells was positively correlated with that of myeloid cells (R^2^ = 0.29, p = 0.00011; **Supplementary Figure 5G**), indicating that these co-enriched myeloid cells may contribute to tumor progression. Thus, we dissected the myeloid subpopulations by integrating their single-cell transcriptomic data from HCC patients (**Supplementary Figure 6A**), and found that the myeloid cells were re-clustered into 22 clusters (**Supplementary Figure 6B**). According to the cluster-specific genes and established myeloid markers, the 22 clusters were divided into eight major subpopulations (**Figure 5A**; **Supplementary Figures 6C, D** and **Supplementary Table 3**).

We also explored the ligand-receptor interactions between the epithelial and myeloid subpopulations using CellPhoneDB (30), and detected significantly more interactions between the MRS2 cells and myeloid subpopulations compared to that among MRS1 cells and myeloid subsets (**Figure 5B**). The MRS2 cells interacted the most with the IL32+, FCN1+ and STMN1+ mono/macrophages (**Figure 5B**). Since these myeloid subpopulations contribute to the immunosuppressive TME (44, 45), we analyzed the type of interactions between MRS2 cells and these myeloid populations, and detected significant enrichment of CD47-SIRPA, TGFB1-TGFβ and other inhibitory ligand-receptor interactions (**Figures 5C–E**). The CD47-SIRPA interaction between tumor cells and myeloid cells is critical to phagocytosis blockage and immune escape (46), which further supports the immunosuppressive role of MRS2 cells in HCC.

We also explored the potential myeloid subpopulations co-enriched with MRS2 cells. The STMN1+ mono/macrophages were positively correlated with MRS2 cells at the single cell and bulk levels (**Figures 5F, G** and **Supplementary Figure 7A, B**). Furthermore, patients with high levels of STMN1+ mono/macrophages showed worse OS (TCGA-LIHC: log-rank test, p = 0.004; GSE14520: log-rank test, p = 0.011; **Figures 5H, I**). Thus, the interaction between STMN1+ mono/macrophages and MRS2 cells *via* SPP1 and CD44 (**Figure 5E**) may result in a persistent immunosuppressive M2 state of myeloid cells (47), leading to the inhibition of immune surveillance. Taken together, the communication between MRS2 cells and myeloid cells shapes the immunosuppressive microenvironment of HCC, resulting in poor clinical outcomes in HCC patients with the MRS2 phenotype.

#### ALDOA is associated with immunosuppressive microenvironment of HCC

**Figure 6:**
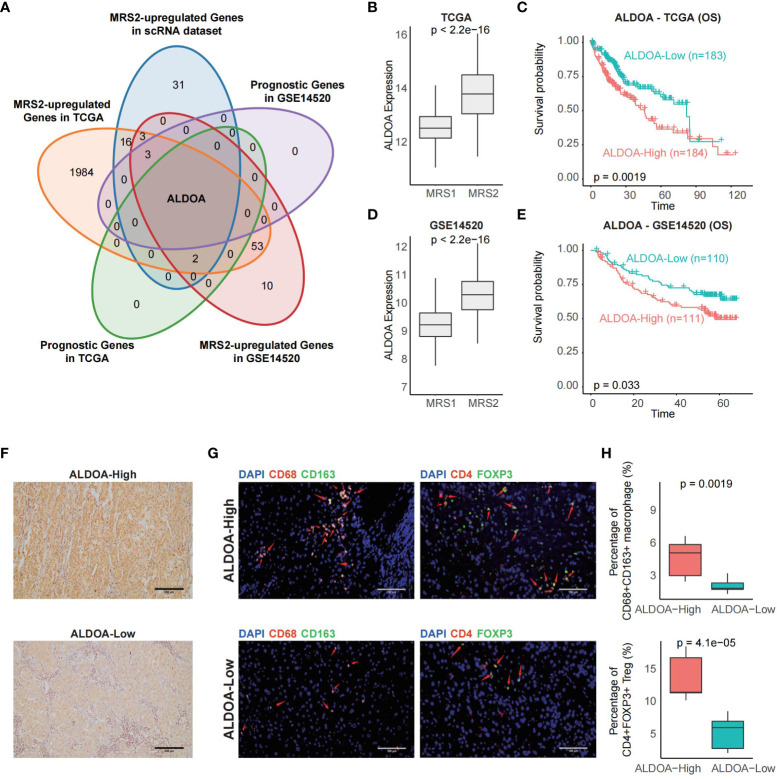
ALDOA overexpression is associated with an immunosuppressive TME **(A)** Venn plot showing the shared up-regulated genes with prognostic relevance in the MRS2-epithelial cells and patients with the MRS2 phenotype. **(B)** ALDOA expression in the MRS1 and MRS2 groups in the TCGA cohort. **(C)** OS of the ALDOA high and ALDOA low HCC patients in TCGA-LIHC cohort. The patients were stratified based on the median expression. **(D)** ALDOA expression in MRS1 and MRS2 groups in the GSE14520 cohort. **(E)** OS of the ALDOAhigh and ALDOA1o HCC patients in GSE14520 cohort. **(F)** Representative images showing low immunostaining of ALDOA in the tumor tissues from ALDOAhigh and ALDOA' groups. **(G)** Left: Representative images of HCC tissues stained with anti-CD68 (red) and anti-CD163 (green) antibodies. Arrows depict the CD68+CD163+ macrophages. Scale bar, 100μm. Right: Representative images of HCC tissues stained with anti-CD4 (red) and anti-FOXP3 (green) antibodies. Arrows depict the CD4+FOXP3+ Treg. Scale bar, 100μm. DAPI (blue) was used to counterstain the nuclei. **(H)** Percentage of CD68+CD163+ macrophages (upper) and CD4+FOXP3+ Tregs (bottom) between ALDOAhigh and ALDOA low groups. Wilcoxon rank-sum test was used to measure the differences between groups.

In order to identify biomarkers of MRS2, we screened for the DEGs between the two metabolic groups, and found that only ALDOA was up-regulated in the MRS2 cells and in patients with MRS2 phenotype (**Figures 6A–E**). Consistent with the prognostic outcomes observed in the MRS2 group (**Figures 3C, F**), patients with higher ALDOA expression had worse OS compared to those with lower ALDOA expression in both TCGA (log-rank test, p = 0.0019, **Figure 6C**) and GSE14520 cohorts (log-rank test, p = 0.033, **Figure 6E**). Immunohistochemical staining also revealed significant differences in ALDOA protein expression between the ALDOA^high^ and ALDOA^low^ groups (**Figure 6F**). Furthermore, ALDOA^high^ patients had a higher fraction of CD68+CD163+ M2 macrophages (p = 0.0019) and CD4+FOXP3+ Tregs compared to the ALDOA^low^ patients (p < 0.0001, **Figures 6G, H**). Thus, immunosuppressive cells are enriched in patients with high ALDOA expression, which may result in poor outcomes.

## Discussion

Cancer cells undergo metabolic adaptations in response to extrinsic and intrinsic stimuli. Some of these adaptations initiate the malignant transformation, while others promote the growth of malignant cells. Most studies conducted on cancer metabolism so far have focused on clinically detectable tumors or phenotypes observed in emergent experimental models. Thus, the terms cancer metabolism and metabolic reprogramming are commonly used to denote a shared set of pathways observed in highly proliferating tumors or cancer cells (48).

Tumor initiation and progression requires the metabolic reprogramming of the malignant cells (20). Furthermore, the endothelial cells, fibroblasts and immune cells in the TME play a key role in promoting tumor progression (49). The cytotoxic T cells (CTLs) that block tumor progression are also inhibited by some bone marrow cells and Tregs to maintain tumor growth (50). We analyzed the single-cell transcriptomics data of multiple HCC datasets in the GEO database (25), and found that epithelial cells were significantly more enriched in HCC patients compared to healthy controls, while the percentage of endothelial cells, T/NK cells and B cells were significantly reduced in HCC patients. Epithelial cell adhesion molecules are biomarkers of cancer stem cells, and can remodel tumors and induce resistance to chemotherapy and radiotherapy. One study showed that epithelial cell adhesion molecules were downregulated by 5-fluorouracil (5FU) in human HCC cell lines (HepG2, Hep3B and HuH-7) and upregulated by cisplatin in the HuH-7 cells, indicating that these molecules are targets of chemoresistance (51) and play an important role in tumor recurrence and progression. Furthermore, the expression levels of APOA2, RPB4, TTR, APOH and some HCC-related genes were significantly different between HCC and control samples (45, 52–54). These findings are consistent with the roles played by different stromal cells during tumor growth (55).

The rapidly proliferating cancer cells use a large amount of glucose to produce lactic acid even under aerobic conditions. This phenomenon is called aerobic glycolysis or Warburg effect (56). However, since glycolysis and TCA cycle are also used by the stromal cells and immune cells, tumor cells must compete with different cell populations in the TME (50). Nevertheless, the conserved metabolic pathways in cancer cells are promising therapeutic targets. Therefore, we also analyzed the expression of metabolism-related genes in the epithelial cells from HCC patients and healthy controls, and detected two distinct metabolic subsets of the epithelial cells from HCC patients based on the predominance of amino acid metabolism (MRS1) and glycolysis/gluconeogenesis (MRS2). Amino acid metabolism plays an important role in tumor progression (57), and pathways involved in tyrosine, glycine, serine, threonine and phenylalanine metabolism, and phenylalanine, tyrosine and tryptophan biosynthesis are activated during the process. In addition, various solid tumor cells die rapidly in medium lacking arginine (58). Likewise, proline dehydrogenase (oxidase) (PRODH/POX), which catalyzes proline to P5C, functions as a tumor suppressor (59). Glycolysis provides carbon intermediates for the biosynthesis of nucleotides, amino acids and lipids, which is essential for the growth of cancer cells (50). HCC patients with a greater abundance of the MRS2 cells had significantly worse survival compared to those with more MRS1 cells. TFF3 is a member of MRS2 and has been noted by researchers in recent years, which promotes tumorigenesis and metastasis by promoting cell proliferation, invasion, metastasis and angiogenesis, and inhibiting cell apoptosis (60).

Most tumor cells express antigens that can be recognized by CD8+ T cells. Therefore, cancer cells have evolved multiple mechanisms to evade anti-tumor immune responses (61). A previous study identified 9 immune-related genes involved in tumor cell proliferation, cell-mediated immunity and tumorigenesis (62). Patients with the MRS2 phenotype exhibited the highest ESTIMATE score, immune score and stromal score, along with the lowest tumor purity. Furthermore, pathways involved in the recruitment of Tregs and MDSCs, which are known to contribute to the immunosuppressive TME (42, 43), were enriched in the MRS2 group. We also identified ALDOA as a biomarker of the MRS2 phenotype, and detected considerable infiltration of M2 macrophages and Tregs in the ALDOA^high^ tumor tissues of the MRS2 patients. Overall, our findings suggested that higher abundance of MRS2 epithelial cells portends worse prognosis due to increased infiltration of the immunosuppressive Tregs and MDSCs.

Myeloid cells, including tumor-associated macrophages and bone marrow-derived suppressor cells, are abundant in the HCC microenvironment and are associated with poor prognosis since they support tumor initiation, progression, angiogenesis, metastasis and drug resistance (63). We found that MRS2 cells rather than MRS1 cells were significantly co-enriched with myeloid cells. In addition, the MRS2 cells and myeloid subpopulations presented significantly more interactions than MRS1 cells and myeloid subsets. A study observed that IL-32 was involved in MRS2, and interacted with STMN1+ mononuclear/macrophage cells, thereby inhibiting tumor development (64). STMN1+ mono/macrophages mediated the immunosuppression *via* interacting with MRS2 cells and resulted in poor clinical outcomes, while the opposite was found for methylation of the gene body region.

To summarize, we identified two distinct metabolic subtypes of HCC that differed in terms of the immunological characteristics of the TME and prognosis, and can be useful for developing targeted therapies. Studies show that there is considerable spatial heterogeneity among cells obtained from the same tissue (65–67), and differences in the enriched cell populations among tumor tissue regions can influence the clinical outcomes of patients (65). The cellular composition of samples used in the scRNA-seq datasets could not reflect the real situation of HCC tissues. Therefore, further analysis is needed to explore the spatial heterogeneity of distinct tissue regions, and identify the predominant subpopulations at different stages of tumor development.

## Data availability statement

The datasets presented in this study can be found in online repositories. The names of the repository/repositories and accession number(s) can be found below: https://satijalab.org/seurat, GSE112271; https://satijalab.org/seurat, GSE149614; https://satijalab.org/seurat, GSE151530; https://satijalab.org/seurat, GSE156625.

## Author contributions

ZH participated in the design of this study, ZH, JC, and SY performed data integration. WH and QH are responsible for obtaining research funds and experimental supervision, and they performed the statistical analysis. ZH drafted the manuscript. QC is responsible for reviewing and revising the manuscript. All authors contributed to the article and approved the submitted version.
