# Supplementary figures and images for: Hepatocellular carcinoma subtypes based on metabolic pathways reveals potential therapeutic targets
Source: Front Oncol. 2023 Mar 2;13:1086604. doi: 10.3389/fonc.2023.1086604 (PMC10017446; doi:10.3389/fonc.2023.1086604)

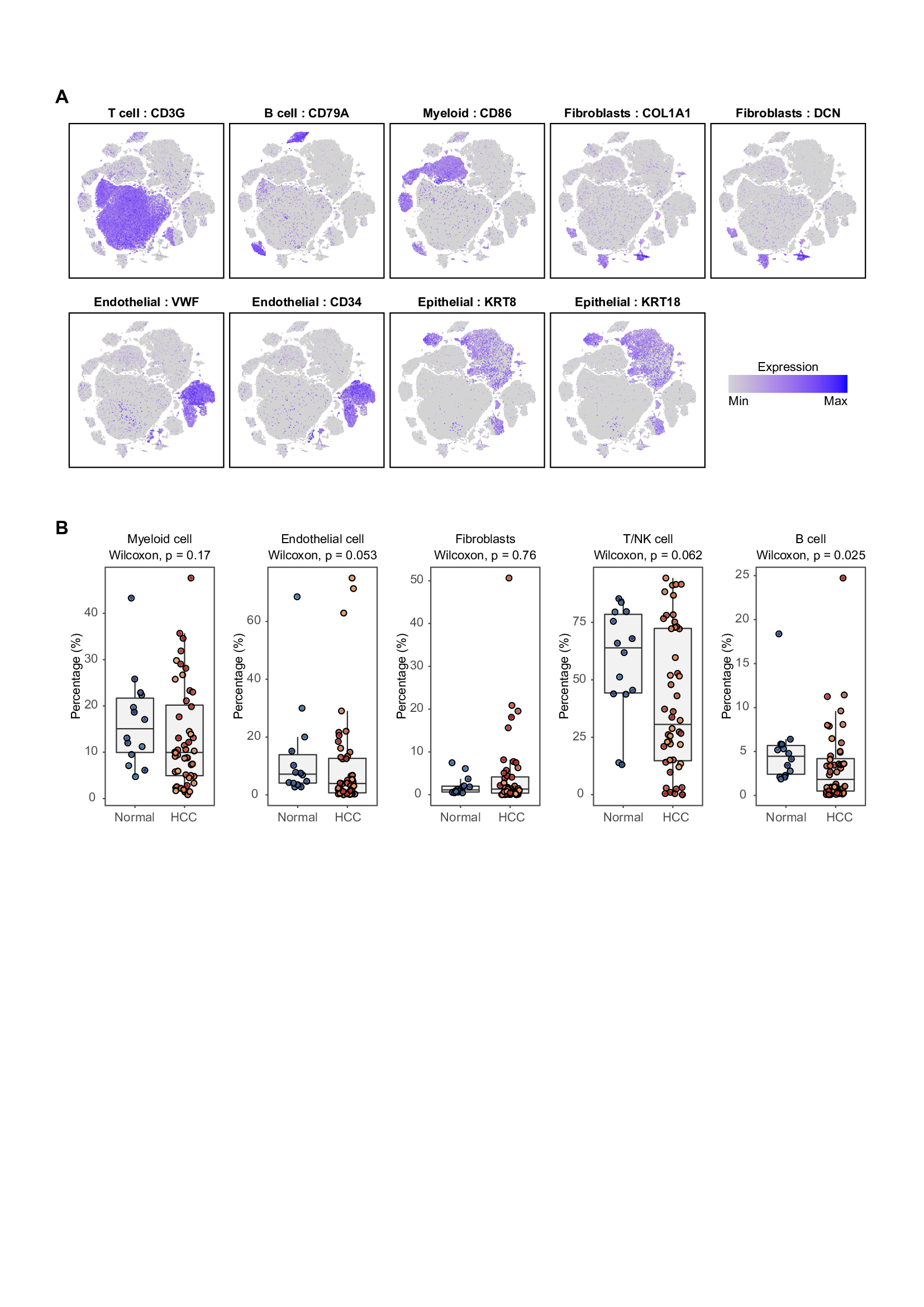

Supplement: Supplementary file 1 [file Image_1.jpeg]

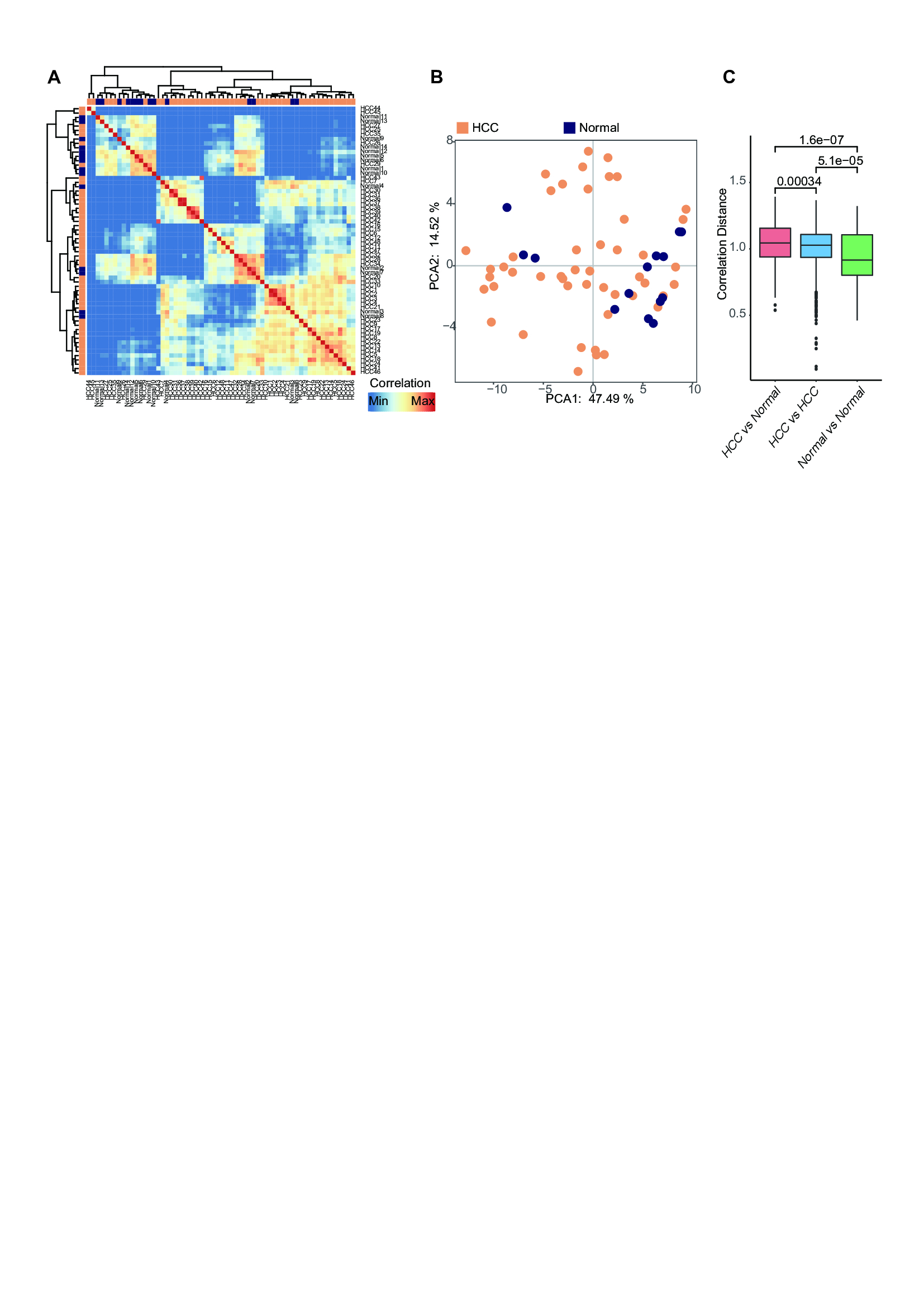

Supplement: Supplementary file 2 [file Image_2.jpeg]

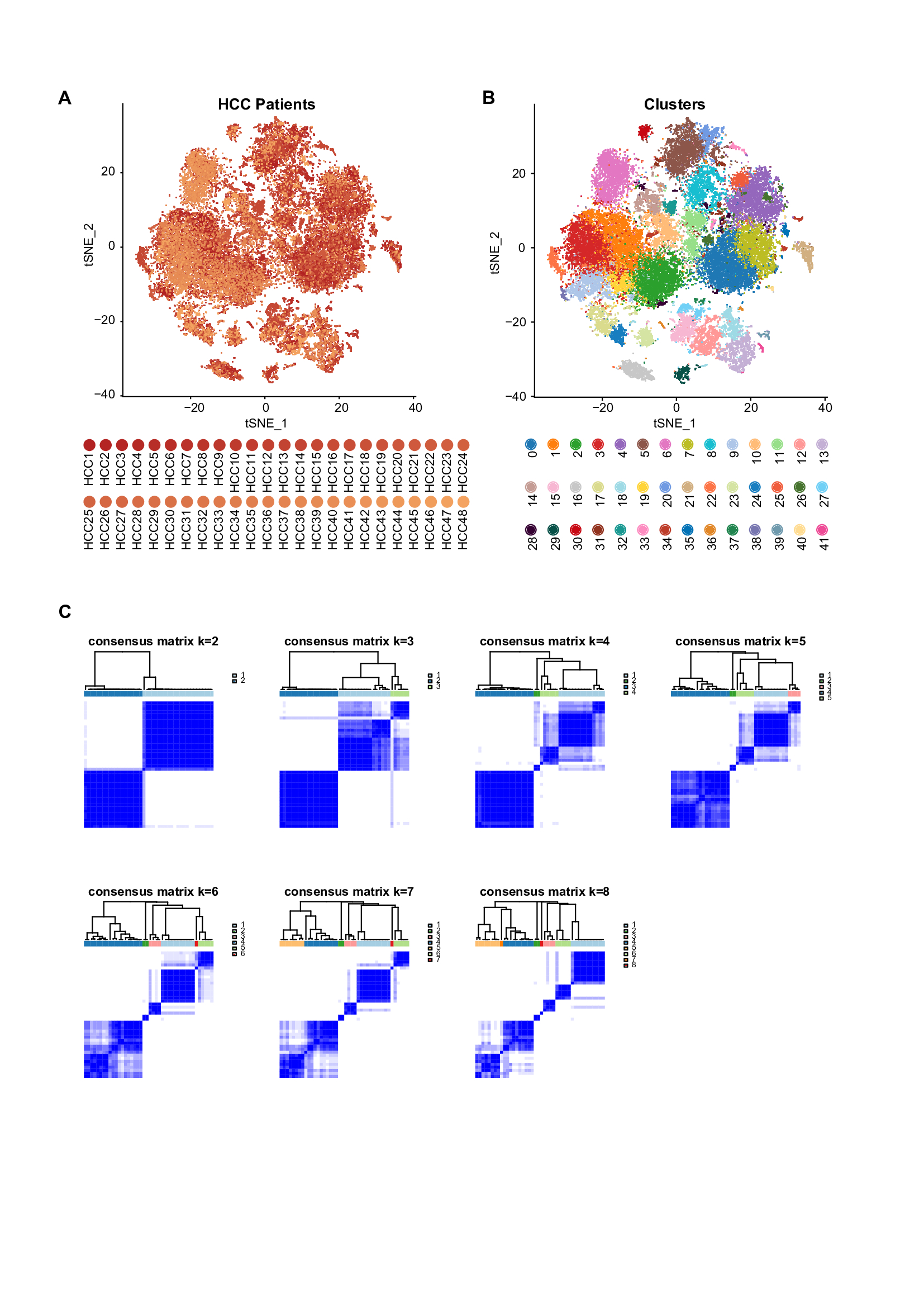

Supplement: Supplementary file 3 [file Image_3.jpeg]

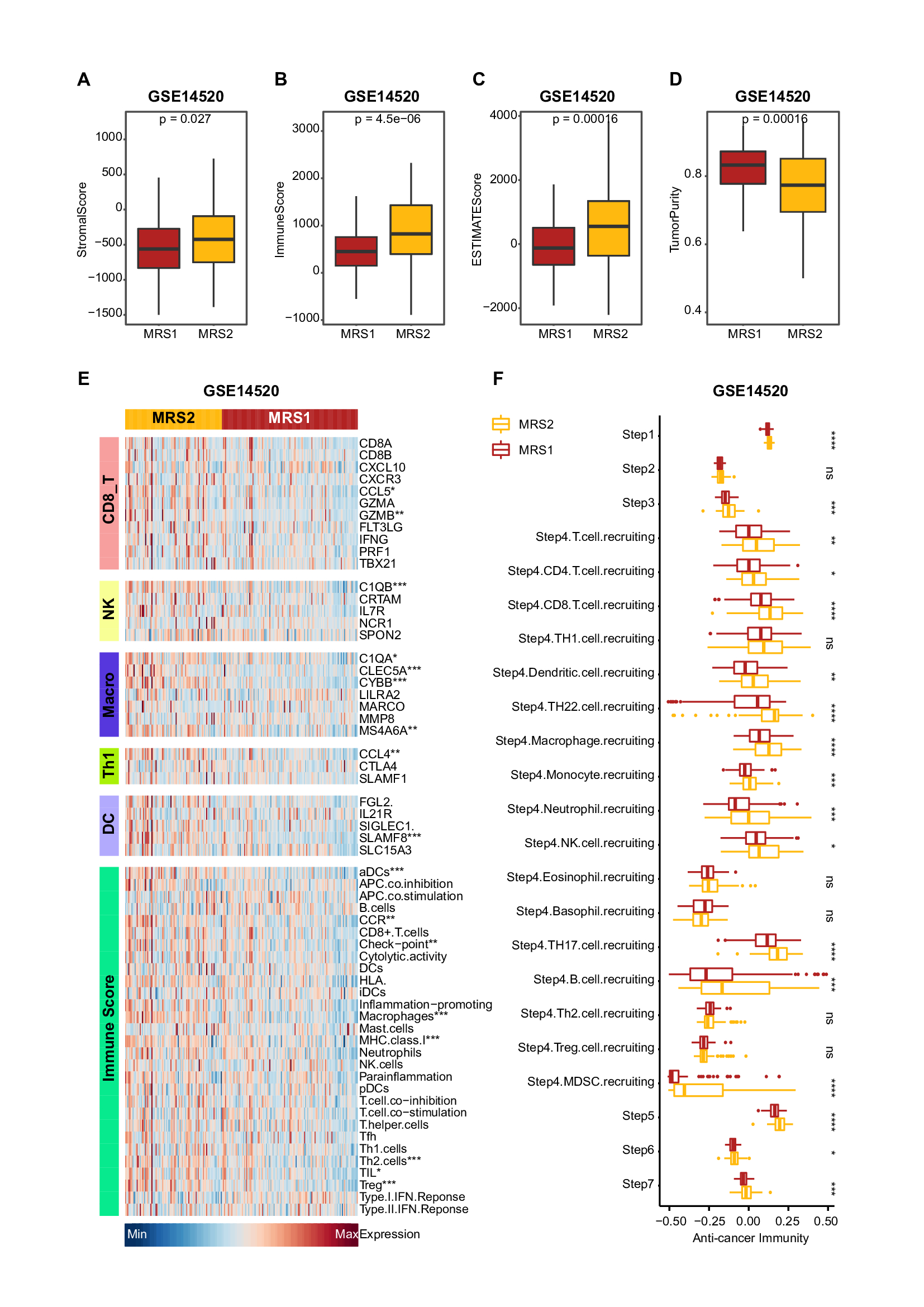

Supplement: Supplementary file 4 [file Image_4.jpeg]

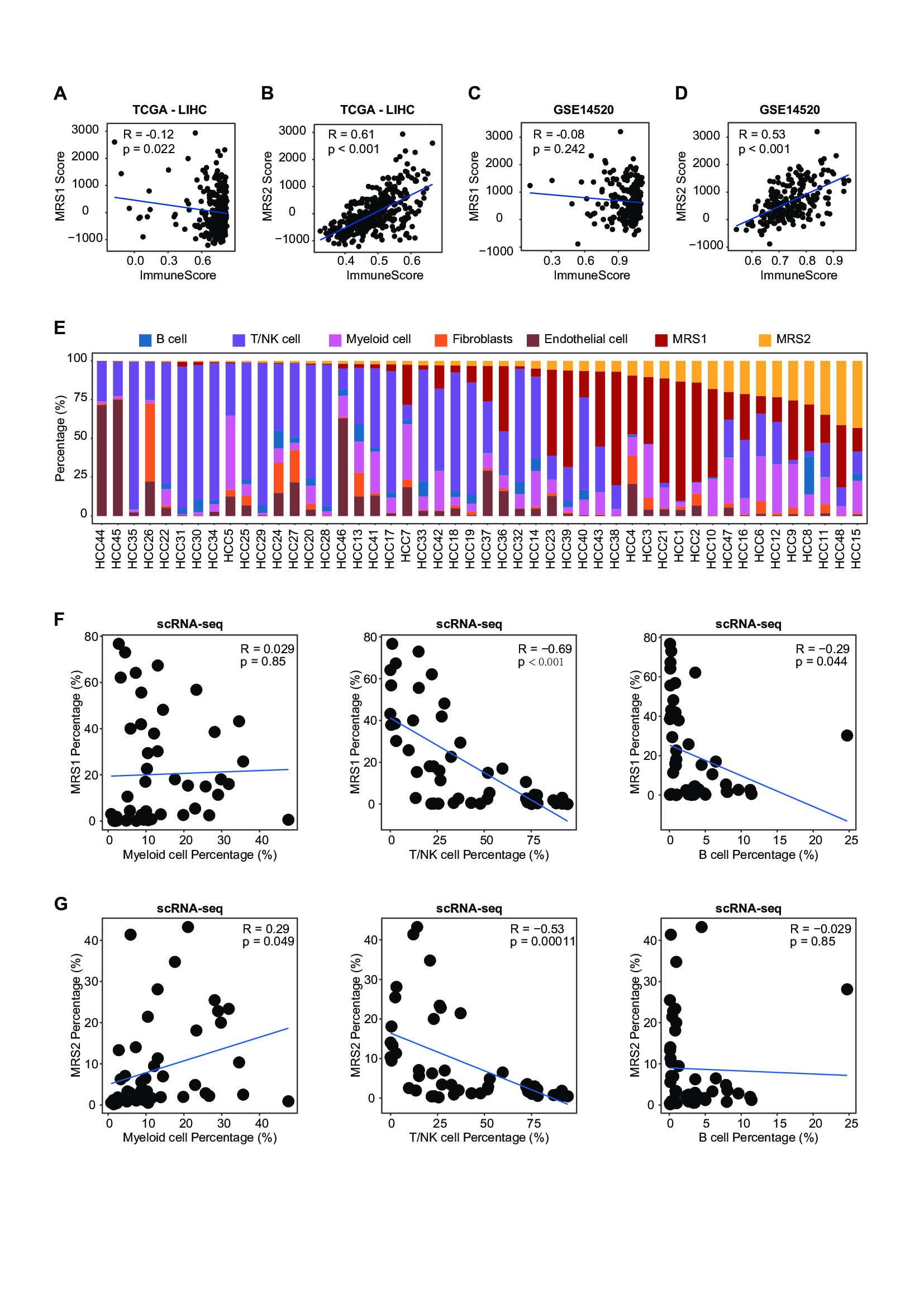

Supplement: Supplementary file 5 [file Image_5.jpeg]

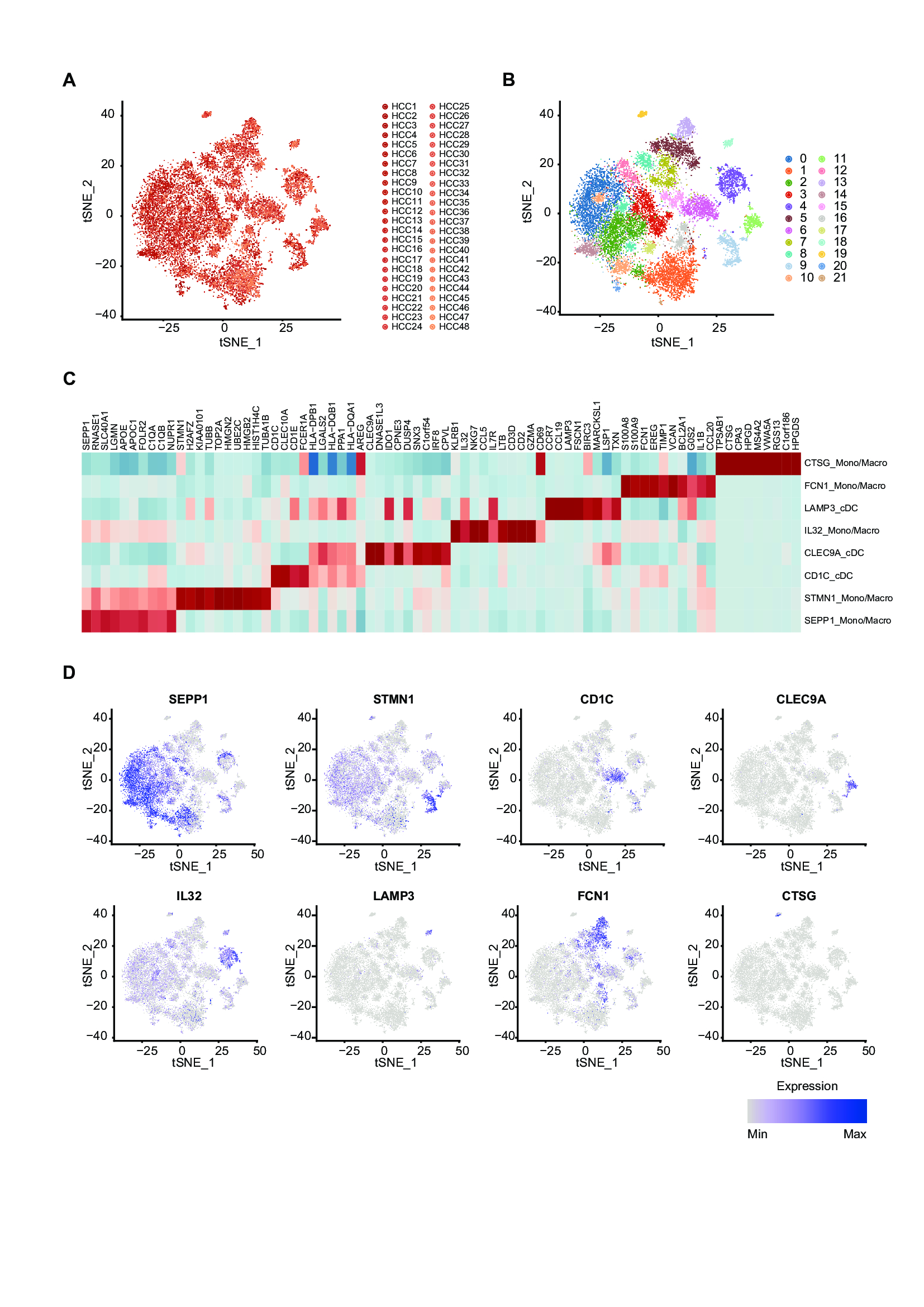

Supplement: Supplementary file 6 [file Image_6.jpeg]

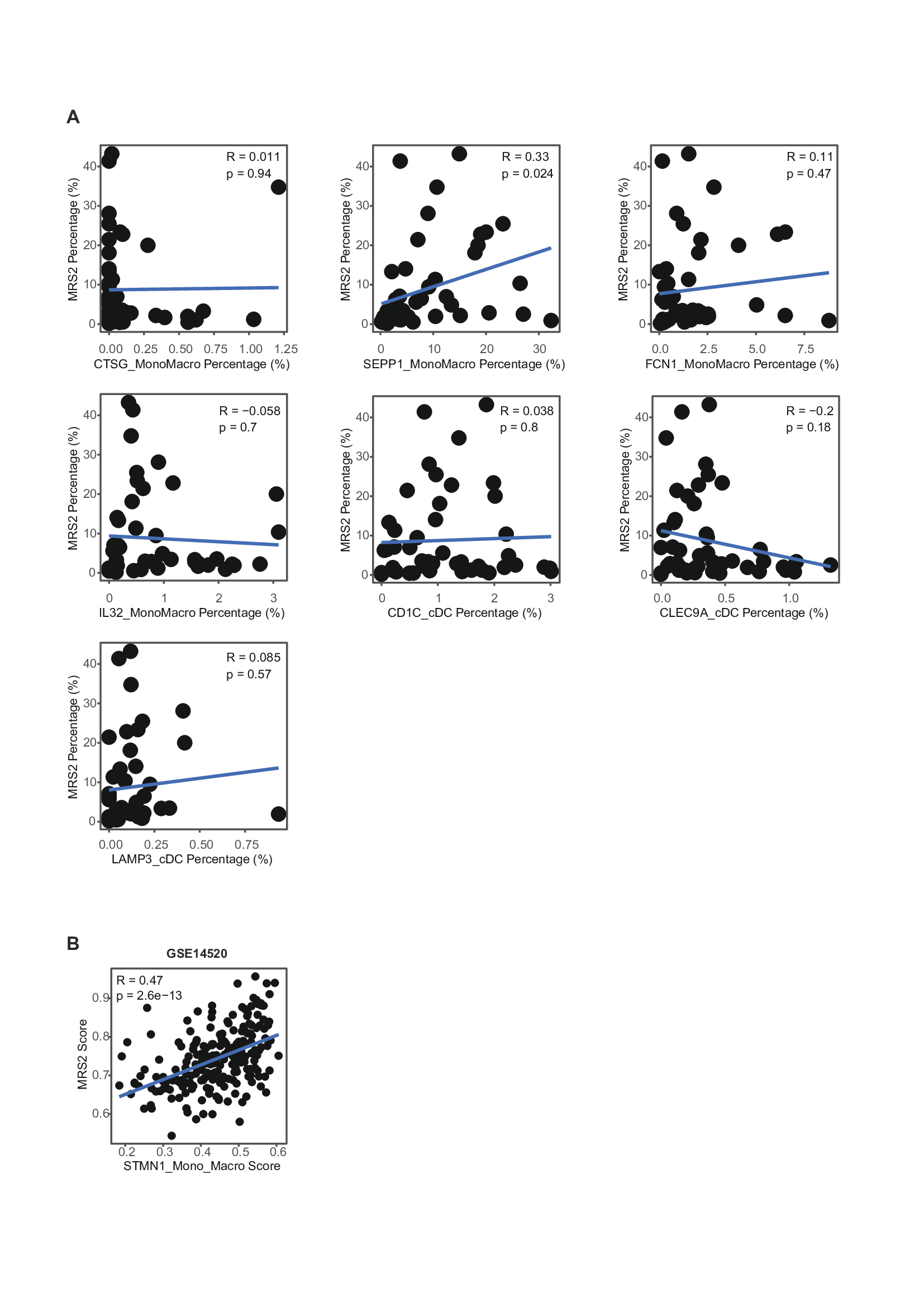

Supplement: Supplementary file 7 [file Image_7.jpeg]
